# Computer simulation study of early bacterial biofilm development

**DOI:** 10.1038/s41598-018-23524-x

**Published:** 2018-03-28

**Authors:** Rafael D. Acemel, Fernando Govantes, Alejandro Cuetos

**Affiliations:** 1 Department of Physical, Chemical and Natural Systems, Universidad Pablo de Olavide, 41013 Sevilla, Spain; 2 Centro Andaluz de Biología del Desarrollo, (Universidad Pablo de Olavide, Consejo Superior de Investigaciones Científicas and Junta de Andalucía), Sevilla, Spain; 3 Departamento de Biología Molecular e Ingeniería Bioquímica, Universidad Pablo de Olavide, Sevilla, Spain

## Abstract

Most bacteria form organized sessile communities, known as biofilms. Their ubiquity and relevance have stimulated the development of efficient mathematical models able to predict biofilm evolution and characteristics at different conditions. Here we present a study of the early stages of bacterial biofilm formation modeled by means of individual cell-based computer simulation. Simulation showed that clusters with different degrees of internal and orientational order were formed as a function of the aspect ratio of the individual particles and the relation between the diffusion and growth rates. Analysis of microscope images of early biofilm formation by the Gram-negative bacterium *Pseudomonas putida* at varying diffusion rates revealed a good qualitative agreement with the simulation results. Our model is a good predictor of microcolony morphology during early biofilm development, showing that the competition between diffusion and growth rates is a key aspect in the formation of stable biofilm microcolonies.

## Introduction

In nature, most bacteria are found as part of sessile communities associated to surfaces and embedded in a polymeric extracellular matrix, designated biofilms^1^. The interest in biofilms derives from their involvement in persistent infectious processes and biodeterioration of materials, but also from their potential applications as efficient biocatalysts for elimination of pollutants or production of chemicals^2,3^. The transition from planktonic to biofilm growth begins with weak, reversible interactions between the bacterial cell and the surface. Commitment to surface growth is marked by stronger, irreversible attachment, followed by loss of flagella when present and clonal growth on the surface to form microcolonies. At this stage synthesis of the biofilm matrix begins. Flat, two-dimensional microcolonies eventually evolve into a mature biofilm featuring complex, three-dimensional structures containing cells immobilized in the biofilm matrix^4,5^. Mature biofilms provide shelter from environmental threats, such as desiccation, predation, or antimicrobial challenge, as well as a stable, nurturing environment that favors metabolic interactions and genetic exchange^6^.

Rod-shaped bacteria generally grow by elongation of their long axes while keeping their width constant. When a bacterial cell has doubled its length, a septum is formed at the equator and the cell is split to form two daughter cells roughly the original size of the mother cell. It is well established that the rate at which bacterial growth and proliferation occurs is driven chiefly by nutrient availability^7^. Bacterial growth by polar elongation of particles is reminiscent of the formation of other complex structures of technological interest, such as the nucleation and growth of calcium-silicate-hydrate particles during cement hardening^8,9^, or the formation of TiO_2_ nanowires^10^. A paramount feature of all of these non-equilibrium processes is the competition between growth and diffusion rates of the particles, due to the fact that polar growth tends to increase the structural order, while diffusion has the opposite effect. Such competition produces structures with complex spatial distributions.

Modeling of biofilm development was previously performed by means of a variety of strategies^11,12^. One of the most promising approaches is when the development of the full biofilm is modeled from the description of the characteristics of the individual bacteria, usually referred as Individual-based Models (IbM)^11–14^. While IbM models commonly represent bacterial cells as spherical particles^11,12^, several instances of models have been developed, mostly very recently, that account for the common rod-like bacterial cell appearance, and the polar growth and critical length-dependent division patterns associated to such geometry^15–20^. As mentioned above, taking these factors into account is likely relevant to explain the forces inside the biofilm that determine the geometrical shape and internal organization of the bacterial colonies.

Here we present a new Brownian Dynamics-based IbM mathematical model for early biofilm microcolony formation. The predictive ability of this model is subsequently assessed by comparing the results of simulation of the time-course of particle cluster growth and organization with equivalent observations of experimental bacterial microcolonies.

### A novel approach to biofilm simulation

In order to improve the strategies used in previous IbM models of biofilm development, we propose an approach in which key features of bacterial cells, such as shape, length, growth rate and passive diffusion rate are considered to model early biofilm growth. For the sake of simplicity, we have introduced the following constraints: (i) the model is limited to the early stages of surface growth, in which the cells divide parallel to the surface, and can therefore be considered a two-dimensional structure; (ii) the model is defined for a submerged biofilm, i.e., a biofilm formed on a surface exposed to a liquid phase, thus allowing brownian motion; (iii) microcolonies are clonal, i.e., the result of the growth and division of an individual cell over time; thus, exchange of cells between the biofilm and the bulk liquid phase is not considered; (iv) active motility in the liquid phase or on the surface is excluded; (v) nutrient concentrations are saturating and nutrient gradients are not significant; therefore the growth rate is constant for all cells; (vi) no changes occur in medium viscosity, temperature or forces binding the cells to the surface, therefore the diffusion rate is also constant; and (vii) no genetic factors modulate the colony organization, i.e., it is determined by purely physical factors.

**Figure 1:**
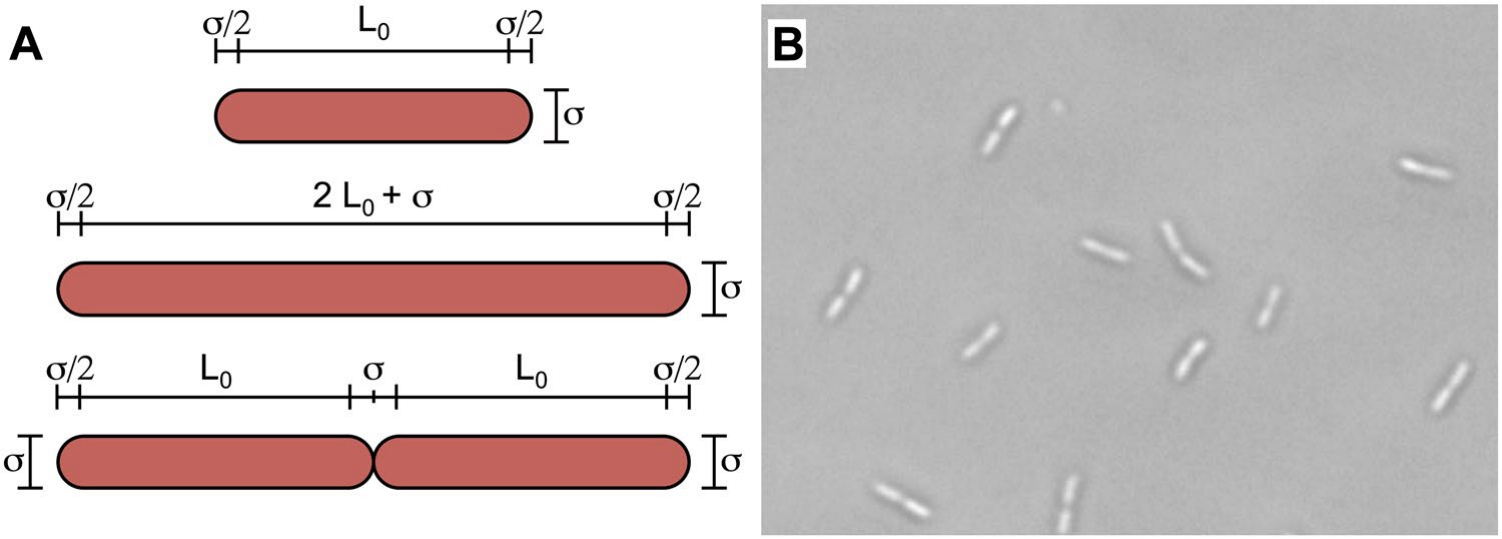
Bacterial cells as spherocylindrical particles. (**A**) Representation of a particle with its initial elongation L_0_, an elongated particle prior to division (L = 2 L_0_ + σ) and two particles immediately after the division (L = L_0_). The main geometric parameters of our model are shown. (**B**) Micrograph of *P. putida* single and dividing cells.

In our model, a post-divisional bacterial cell is represented by a soft spherocylindrical particle^21^ with an initial length L_0_ + σ and diameter σ. Accordingly, the initial aspect ratio of the particles is L*_0_ = L_0_/σ + 1 (Fig. 1A). The particle grows by polar elongation and increases its length over time at a constant velocity v_gr_ while keeping its width σ constant. When the particle reaches its maximum aspect ratio L*_m_ = 2 L*_0_ it is split transversally into two identical particles with am initial aspect ratio of L*_0_ and the same orientation as the parent particle (Fig. 1A). Biofilm cells in general lack flagella^22^, and assuming that surface-linked motility mechanisms are also absent, cells may be displaced by contact with other cells or by brownian diffusion. We have chosen to simulate this scenario using Brownian Dynamics^23^. In this dynamics, particle diffusion is modulated by the diffusional coefficient D_0_, which depends on temperature, the adhesion force between particle and surface and medium viscosity. Considering all the elements described up to now, in the framework of our model the evolution of a biofilm along time is controlled by L*_0_, v_gr_ and D_0_. To integrate the opposite effect of growth and diffusion, we have defined the combined parameter Г = t_dif_/t_gr_, where t_dif_ is the average time required for an isolated particle of constant aspect ratio L*_0_ to be displaced a distance σ by means of brownian diffusion, and t_gr_ is the time required for a particle to reach the aspect ratio L*_m_ from its initial aspect ratio (see Methods section below for details). Preliminary analyses indicated that results of the simulation are dependent on the value of Г, independently of the actual values of v_gr_ and D_0_.

### Competition between diffusion and elongation reveals two distinct cluster growth regimes

In order to model the early stages of bacterial biofilm development, we carried out simulations with particles with an initial aspect ratio L*_0_ = 2.6, which is consistent with the experimentally determined initial aspect ratio of the Gram-negative bacterium *Pseudomonas putida* (Fig. 1B), used below for experimental validation of the model. In addition, as the physics of non-equilibrium processes where the competition between diffusion and polar elongation with division may be relevant to other fields of technological interest, we have extended our study to particles with greater aspect ratios (up to L*_0_ = 6) that may not correspond to realistic bacterial cells. Similarly, although our experimental results show that colonies containing more than 128 cells lose the two-dimensional character, we have extended our study to larger numbers (up to 1024) of particles.

**Figure 2:**
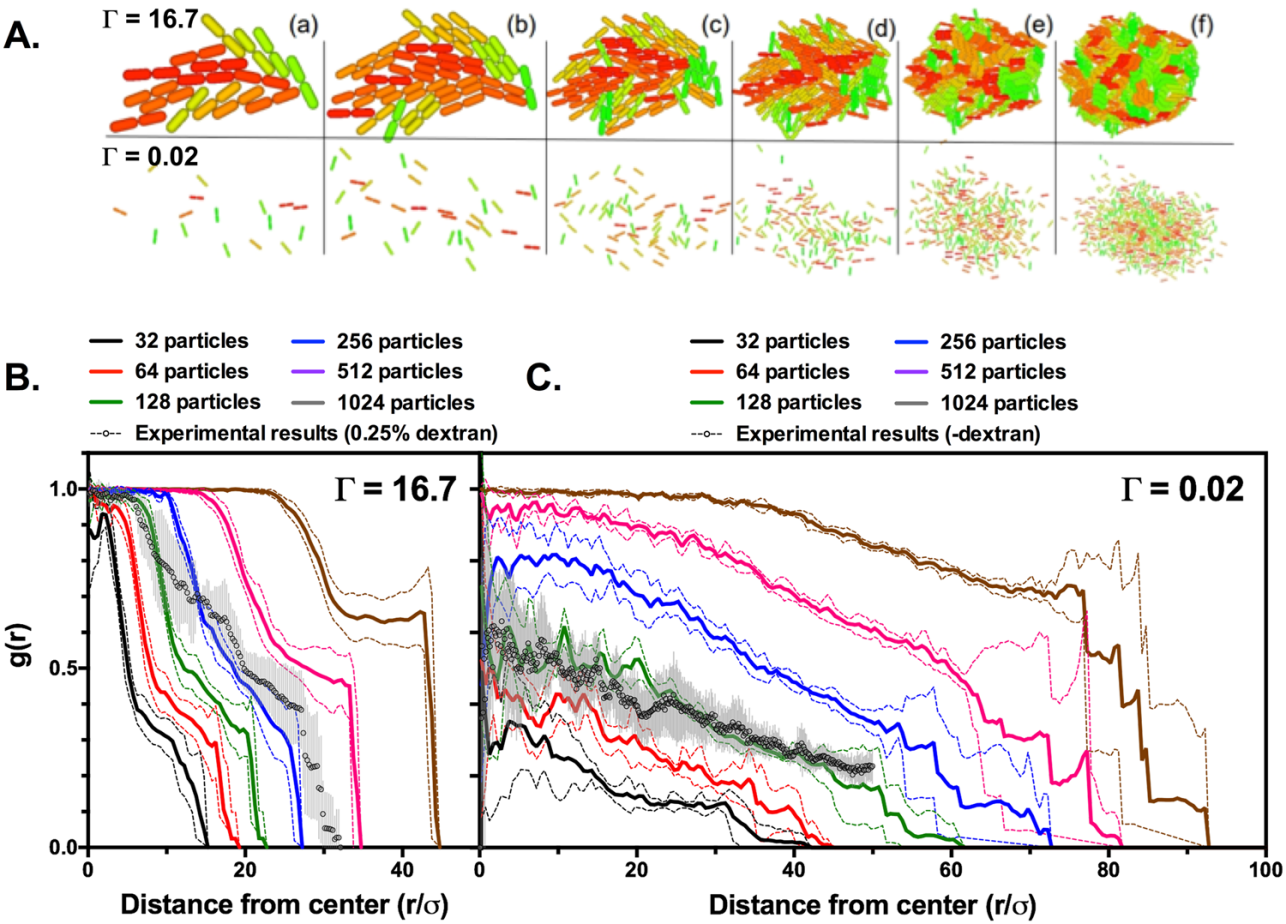
Surface coverage of simulated particle clusters and biofilm microcolonies. (**A**) Snapshots of 32, 64, 128, 256, 512 or 1024 (a–f) particle clusters with L*_0_ = 2.6, and Г = 16.7 (top) or 0.02 (bottom). B. and C. Surface coverage profiles g(r) of clusters with L*_0_ = 2.6, and Г = 16.7 (**B**) or 0.02 (**C**). Clusters containing 32, 64, 128, 256, 512 or 1024 particles are denoted by black, red, green, blue, purple and grey lines, respectively. Circles denote surface coverage profiles obtained from MRB52 microcolonies in the presence (**B**) or in the absence (**C**) of 0.25% dextran sulfate. Lines and data points represent the average of 10 independent simulations or 10 independent microcolonies containing 117 ± 11 and 65 ± 10 (for the experimental results displayed in B and C, respectively). Dashed lines (simulation data) and error bars (experimental data) represent the standard deviation of the mean.

Visual inspection of snapshots depicting the time-course of particle cluster configurations obtained from computer simulations revealed two distinct growth regimes, governed by the values of L*_0_ and Г (Fig. 2A). High Г values, in which growth predominates over diffusion, favored the growth regime designated here as “closed growth”, in which low diffusion of particles between divisions led to the formation of compact clusters made out of tightly packed particles. This behavior was also more prominent at high L*_0_ values. In contrast, low Г values favored the growth regime designated here as “open growth”, in which fast diffusion of particles led to the formation of larger, looser clusters in which spaces were progressively filled by the products of subsequent particle growth and division events. This behavior was also more prominent at low L*_0_ values. The coverage profile g(r) is defined as the fraction of surface covered by particles at distance r from the mass center of the cluster, with values ranging from 0 (no surface coverage) to 1 (total surface coverage) (see Methods section below for details). We have used g(r) to characterize the two growth regimes described above (Fig. 2B,C). Analysis of the evolution of g(r) over time in clusters obtained from simulations at L*_0_ = 2.6 and Г = 16.7, displaying closed growth (Fig. 2B), revealed an area of very high coverage (g(r) >0.9) around the center that was evident very early in cluster development (at the 32-particle stage) and expanded outward over time. This area was limited by a sharp decline of g(r) at its edge. These observations are consistent with the notion that closed growth clusters are composed of tightly packed particles covering most of the surface within the cluster. Such clusters expand preferentially at their outer edge with no significant change in their degree of internal compactness. In contrast, analysis of the evolution of g(r) over time in clusters displaying open growth (L*_0_ = 2.6, Г = 0.02)(Fig. 2C) showed an area with low g(r) values (<0.5) around the center of the clusters at the 32-particle stage. The g(r) values declined progressively with distance from the cluster center, but the cluster major radius (defined by the maximum distance at which g(r) >0) was significantly (>2-fold) greater than that observed in the open growth condition at this stage, consistent with the notion that open growth clusters are arrays of loosely arranged particles spanning a large area. Cluster growth was evidenced by a progressive increase in both g(r) and major radius over time, thus lending support to the hypothesis that open growth is characterized by simultaneous expansion at the outer edge of the cluster and filling of the spaces between particles by growth and division of particles within the cluster.

### L*_0_ and Г determine the cluster shape and orientational organization

**Figure 3:**
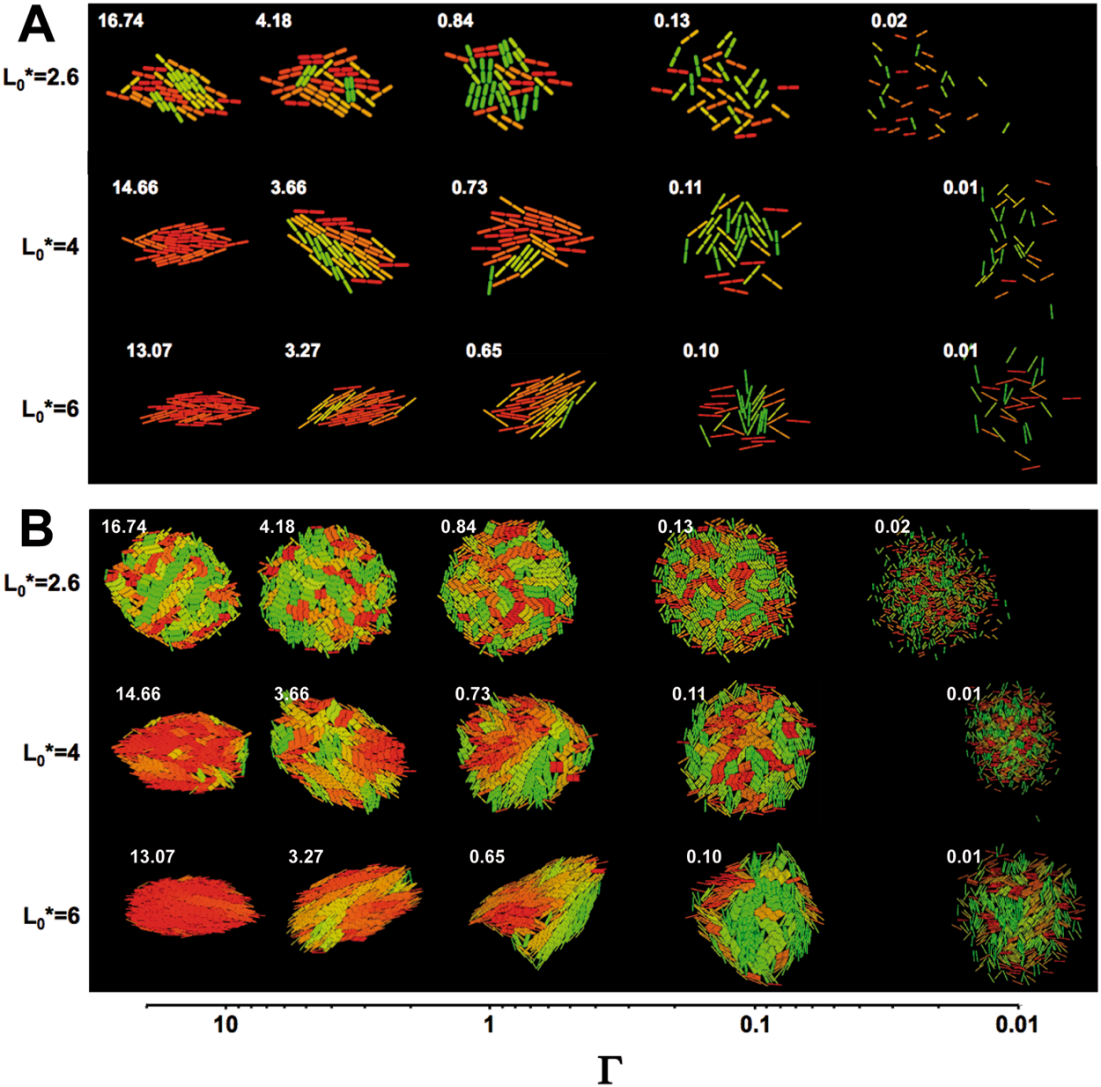
Typical results from computer simulations. Snapshots showing the shape and internal structure of clusters containing 64 (**A**) or 1024 (**B**) particles with aspect ratios L*_0_ = 2.6, 4 or 6 and variable Г values. Particle color indicates orientation in a scale ranging from green (vertical) to red (horizontal).

We have explored further the structural features of particle clusters as a function of the L*_0_ and Г parameters. Fig. 3 and Supplementary Figure S1 show snapshots depicting the outcome of a typical simulation experiment. Characteristic configurations of 64- (Figs 3A and S1A) and 1024-particle (Figs 3B and S1B) clusters immediately after division under a variety of L*_0_ and Г values are displayed. Visual inspection of these results evidences a strong influence of L*_0_ and Г on the shape and internal structure of the clusters. Accordingly, for L*_0_ = 6, at the highest Г values tested (Г ≈ 10^1^) the clusters were highly compact, with an ellipsoidal shape with individual particles almost perfectly aligned resembling a nematic phase in the orientation of the major axis of the cluster. On the other hand, for low Г values (Г ≈ 10^−2^) the clusters displayed a looser structure with a quasi-circular shape and the particles were not greatly biased in their orientation, as shown by the occurrence of multiple small nematic-like domains. At intermediate values of Г the shape of the clusters became more irregular, transitioning through progressively less eccentric ellipsoidal shapes as the Г values decreased. Similarly, the level of internal organization diminished, as shown by the increase in number and decrease in size of the nematic domains with decreasing Г values. A similar trend was observed at lower L*_0_ values, although in general the shorter aspect ratios appeared to favor looser clusters with scarce orientational organization.

**Figure 4:**
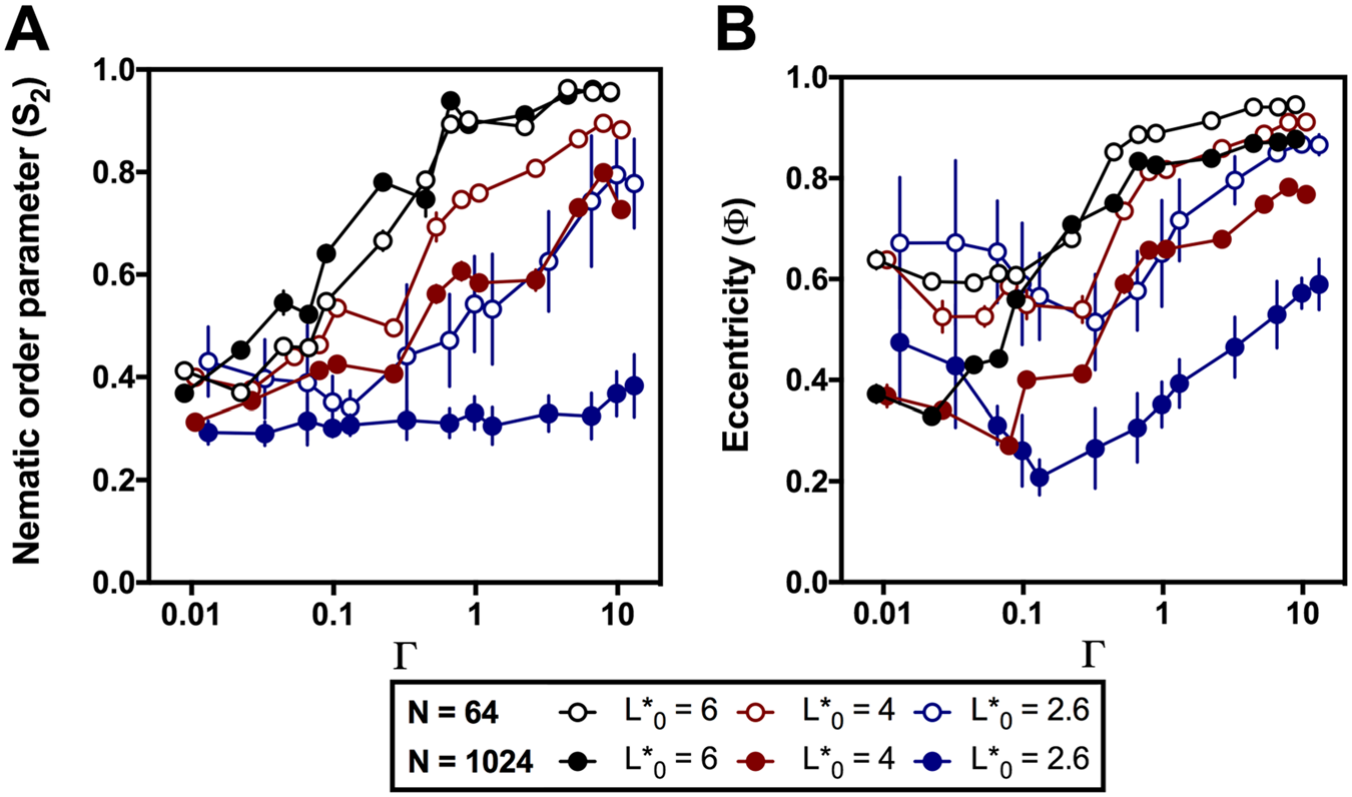
Measurement of the nematic order parameter (S2) and eccentricity (Φ) in simulations. (**A**) Dependence of the nematic order parameter S_2_ with Г. (**B**) dependence of the eccentricity Ф with Г. Both plots display simulation results for clusters containing 1024 particles (solid symbols) or 64 particles (open symbols), containing particles with L* = 6 (black), 4 (red) or 2.6. Error bars represent standard deviation of the mean, and are only shown when larger than the corresponding symbol.

To characterize further the qualitative behavior described from the visual inspection of the images obtained from the simulations, the evolution of the nematic order (S_2_)^24,25^ and eccentricity (Ф) parameters relative to the Г values was examined for the 64 particle and 1024 particle datasets at L*_0_ = 2.6, 4 and 6 (Fig. 4) (for details, see the Methods section below). The values of S_2_ range from 0 if the orientation of the particles is completely random, to 1 if all the particles are arranged in the same orientation. The results showed in Fig. 4A indicate a clear positive correlation between the S_2_ and Г values for different number of particles and values of L*_0_, thus confirming the observation above that increasing Г values promote the occurrence of clusters with increasing orientational organization. In addition, a positive correlation was also observed between orientational organization and aspect ratio, as shown by the fact that S_2_ values were highest for clusters with L*_0_ = 6 and lowest for those with L*_0_ = 2.6 over a wide range of Г values. Finally, it is worth noting that 64-particle clusters only showed this behavior at Г values greater than 0.1, while relatively low S_2 values_ that were not significantly influenced by Г were observed at Г values below 0.1. Clusters with L*_0_ = 2.6 simulated at large Г values also demonstrated a remarkable evolution in their internal organization over time, as the clear, Г-dependent increase of S_2_ observed at the 64-particle stage resulted in low, Г-independent S_2_ values at the 1024-particle stage, indicating that regardless of the level of orientational order present in the early stages, clusters made of particles with L*_0_ = 2.6 become highly disordered as the number of particles increases. In contrast, comparison of 64- and 1024-particle clusters only revealed modest changes at L*_0_ = 4 and no significant changes at L*_0_ = 6. Taken together, our results confirm the observation that predominance of growth over diffusion and large aspect ratios promote a high level of internal organization in which particles are arranged in similarly-oriented domains, while predominance of diffusion over growth and small aspect ratios favor the random organization of particles within the clusters. In addition, we provide evidence that short particle clusters show a tendency to decrease their internal organization as they increase in size, while long particle clusters tend to maintain a similar level of organization over time.

Similarly, eccentricity (Ф) was used to assess the deviation of particle clusters from a circular shape. Eccentricity values range from 0 for a perfect circle to close to 1 for highly eccentric elliptic shapes. As shown in Fig. 4B, eccentricity showed a non-monotonous behavior relative to Г: at high Г values, Ф increased with increasing Г. However, at low Г values, Ф increased with increasing Г. The Г value showing the minimum eccentricity was greater at lower aspect ratios and in the 64-particle compared to the 1024-particle series. This result correlates very well with the initial observation of two distinct growth regimes: under conditions promoting closed growth conditions eccentricity increased with ГГ, likely due to interference of neighboring particles with particle rotation in the X-Y plane within the tightly packed clusters. In contrast, conditions promoting open growth initially showed the opposite pattern, as the low coverage (e.g., at the 64-particle stage) prevented such interference, but mimicked the behavior of the closed growth regime when high surface coverage (e.g., at the 1024-particle stage) enabled interference on rotation. Under high surface coverage conditions, the behavior of eccentricity was reminiscent of that of the nematic order parameter; i.e., it increased with increasing aspect ratio and decreased with cluster size. Our results confirm the general observation that high L*_0_ and Г values promote the formation of clusters with high surface coverage, internal organization and eccentricity, while low L*_0_ and Г values promote the formation of clusters with low initial surface coverage that increases progressively with time, and low internal organization, while the positive correlation between Г and eccentricity is observed prominently when clusters have reached a sufficient size to display high values of surface coverage.

### Experimental validation: early biofilm development in *Pseudomonas putida*

**Figure 5:**
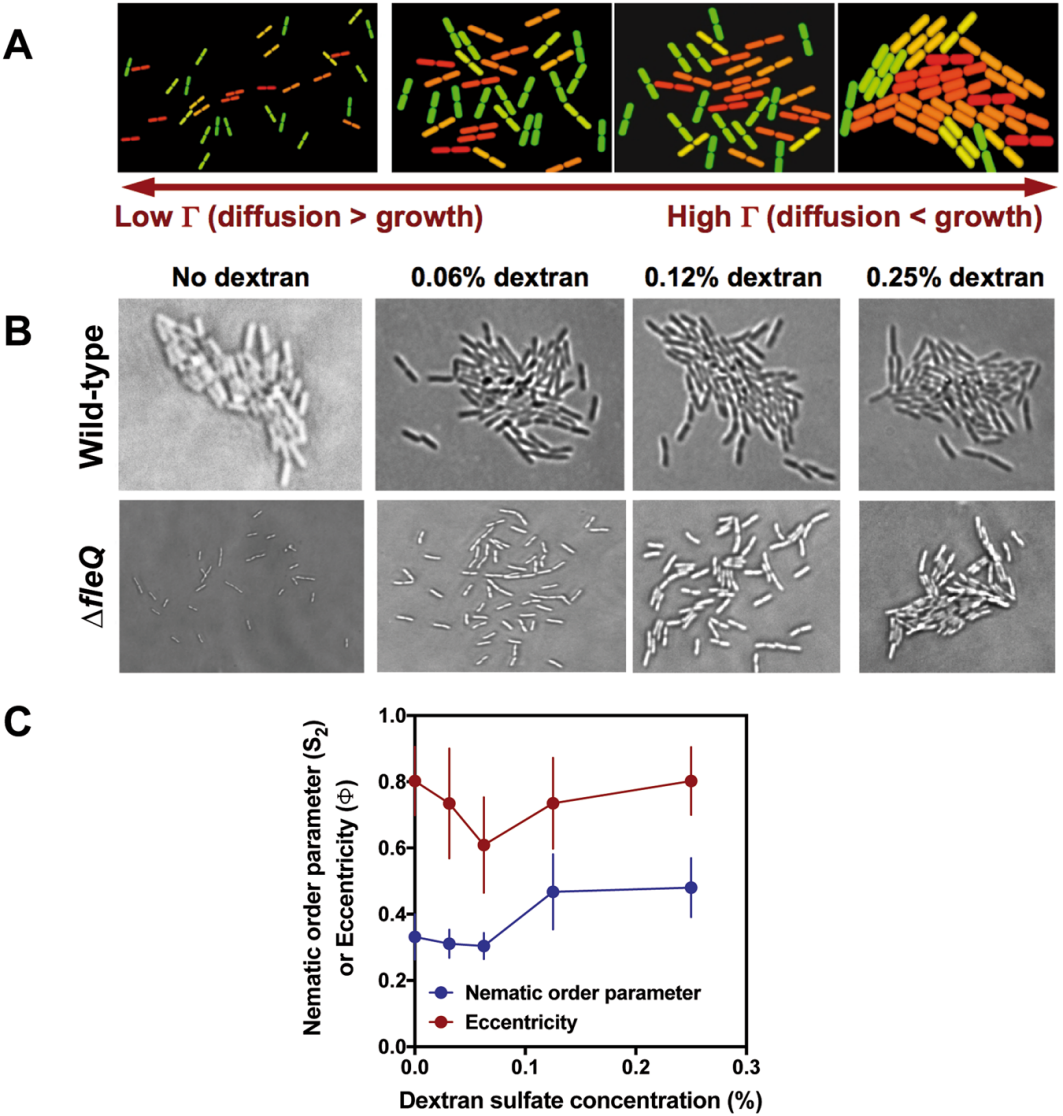
Comparison of simulation and live bacterial microcolony microscopy results. (**A**) Typical snapshots of simulated clusters containing 64 particles of L*_0_ = 2.6 at different Г values. (**B**) Micrographs of microcolonies containing ~70 cells of the wild-type and ∆*fleQ P. putida* strains KT2442 and MRB52 at different dextran sulfate concentrations. (**C**) Plot representing the experimentally derived eccentricity (Ф) and nematic order parameter (S_2_) values for colonies of MRB52 grown at different dextran sulfate concentrations. Symbols represent the averages obtained from ten colonies containing 65 ± 9 cells (no dextran sulfate), 74 ± 11 cells (0.031% dextran sulfate), 112 ± 17 cells (0.062% dextran sulfate), 91 ± 19 cells (0.125% dextran sulfate), and 117 ± 8 cells (0.25% dextran sulfate), respectively. Error bars represent standard deviation of the mean.

We have tested the validity of the simulation described above as a predictor of biofilm microcolony structure and growth by microscopic imaging of submerged biofilm microcolonies of the Gram-negative bacterium *P*. *putida*. *P*. *putida* cells are rod-shaped, with an experimentally determined initial aspect ratio of 2.59 ± 0.15. In our experimental conditions, attachment of wild-type *P*. *putida* cells to the substrate is followed by proliferation on the surface plane for ~6–7 cell divisions before the colonies become three-dimensional (see Supplementary movie S1). Because flagellar motility is inhibited upon attachment, diffusion is determined by the strength of attachment and the ability of the cells to diffuse in the liquid phase by brownian motion. Two *P*. *putida* strains were chosen for this purpose, the attachment-proficient wild-type strain KT2442, and its attachment-deficient non-motile mutant derivative MRB52, carrying a deletion of the *fleQ* gene, encoding the master regulator of flagellar biogenesis and biofilm formation^26^. Visual inspection of microcolonies of KT2442 and MRB52 strains revealed two distinct behaviors: while the wild-type strain formed small, compact and highly eccentric microcolonies with a high degree of orientational organization, the ∆*fleQ* strain formed large, loose colonies with less eccentricity and poor conservation of the orientation among single cells (Fig. 5B; “No dextran” panels). These results suggest that the strong surface attachment displayed by the wild-type strain limits brownian diffusion, resulting in a microcolony structure reminiscent to the clusters observed in the simulation with particles of L*_0_ = 2.6 at high Г values (Fig. 3A). In contrast, weak interaction with the surface in the ∆*fleQ* mutant likely results in predominance of diffusion over growth, hence the structural similarity between the MRB52 colonies and the particle clusters formed in the simulation with particles of L*_0_ = 2.6 at low Г values (Fig. 3A). The time-course of surface proliferation of MRB52 is shown in Supplementary movie S2.

**Figure 6:**
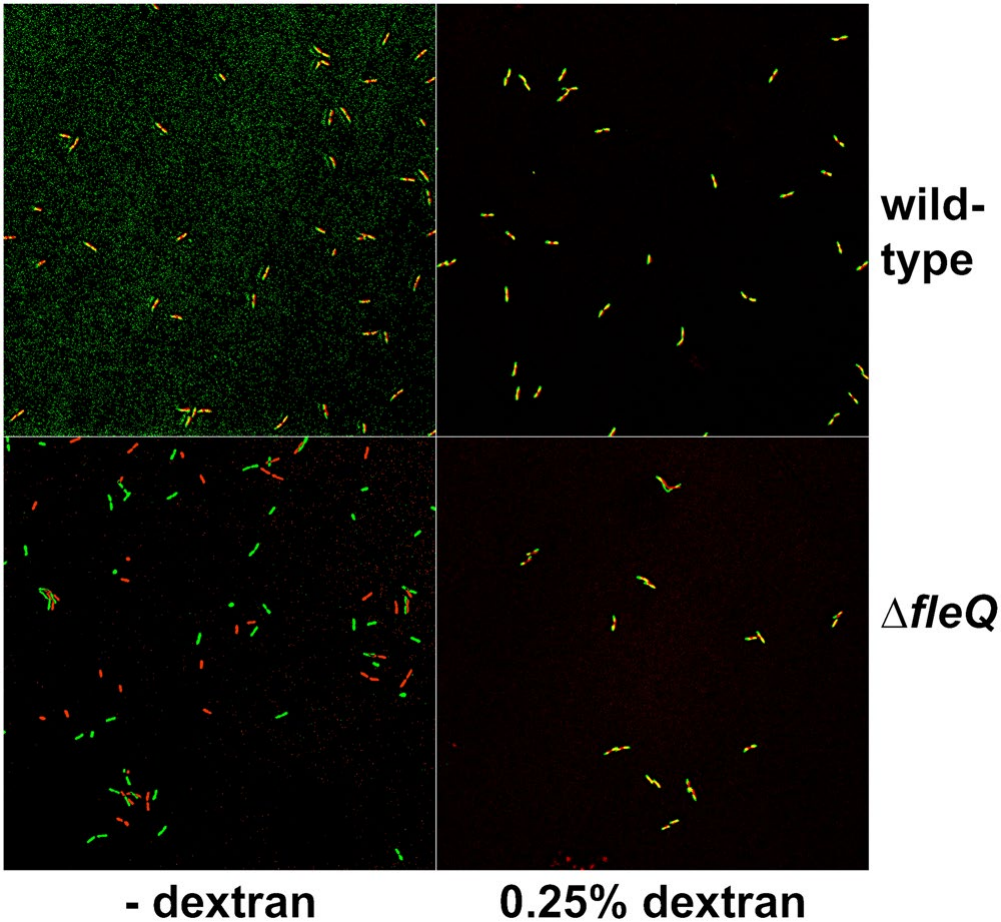
Effect of dextran sulfate on brownian diffusion of individual wild-type and ∆fleQ P. putida cells. Images are overlays of artificially colored surface-associated wild-type (**A**) and ∆*fleQ* (**B**) cells showing their initial (time = 0; green) and final positions (time = 10 minutes; red). Overlapped positions of motionless cells are indicated in green. Note that overlap may not be perfect due to cell growth during the 10-minute incubation. The complete time-lapse sequences are available as Supplementary videos 1–4.

In order to manipulate Г in our experimental setup, we performed a new set of experiments in which different concentrations of the polymer dextran sulfate were added to the growth medium. Dextran sulfate-containing solutions display a concentration-dependent increase in viscosity^27^, which, according to our model, is predicted to provoke an increase in the Г value. To assess the possible effect of dextran sulfate on brownian motion of individual cells we used time-lapse microscopy to monitor the position of wild-type and ∆*fleQ* cells that had been allowed to attach to the surface for only five minutes, in the presence and in the absence of dextran sulfate (Fig. 6 and Supplementary movies S3–S6). Brownian motion was barely detectable for the wild-type cells regardless of dextran sulfate addition, suggesting that the strong attachment of this strain even after this short time period limits cell diffusion in the XY plane (Fig. 6A, and Supplementary movies S3–S4). Interestingly, the ∆*fleQ* strain displayed clear brownian diffusion in unsupplemented medium, but this phenomenon was suppressed by the addition of 0.25% dextran sulfate (Fig. 6B, and Supplementary movies S5–S6), supporting the notion that increased viscosity limits diffusion of the attachment-deficient strain.

Next, we examined the effect of different dextran sulfate concentrations on the morphological features of biofilm microcolonies. Visual inspection of micrographs taken from typical microcolonies of the wild-type strain failed to show any significant dextran concentration-dependent changes in microcolony morphology and structure (Fig. 5B), consistent with the notion that strong surface attachment is highly detrimental to diffusion by brownian motion. In contrast, a dextran concentration-dependent transition from large, loose, disorganized colonies to small, compact and highly ordered colonies was evident in the ∆*fleQ* mutant (Fig. 5B). Remarkably, this behavior faithfully replicated the transition observed in the clusters obtained in the simulation with particles of L*_0_ 2.6 at increasing Γ values (Fig. 5A), indicative of a strong correlation between the prediction of the simulation experiments and the experimental behavior of the biofilm microcolonies.

To characterize further the effect of increased viscosity on microcolony structure, we determined the surface coverage profile of MRB52 microcolonies grown with no dextran sulfate (Fig. 2C) or in the presence of 0.25% dextran sulfate (Fig. 2B). The colonies grown with no addition (65 ± 10 cells per colony) displayed relatively low surface coverage at their center, and the coverage values decreased gradually toward the colony periphery to span a relatively long distance from the center. This behavior closely mimics the surface coverage profiles obtained with the simulation at a low Г value (Fig. 2C), strongly suggesting that development of the ∆*fleQ* mutant microcolonies under low viscosity conditions follows an open growth regime as defined above. In contrast, colonies grown with 0.25% dextran (117 ± 11 cells per colony) displayed nearly full surface coverage in a narrow area around the center of the colony that was limited by a sharp drop in coverage. This behavior is reminiscent of the surface coverage profiles obtained at a high Г value (Fig. 2B). Taken together, these results strongly suggest that increased viscosity induces a shift in the ∆*fleQ* mutant microcolony development from an open growth to a closed growth regime. The remarkable correlation between the coverage profiles obtained in the simulations at low and high Г and those obtained from microscopic images of bacterial microcolonies in low and high viscosity media, respectively, indicates that our mathematical model accurately predicts this viscosity-dependent shift in the microcolony growth regime.

Finally, we also calculated the eccentricity (Ф) and the nematic order parameter (S_2_) for microcolonies of the ∆*fleQ* mutant MRB52 grown under a variety of dextran sulfate concentrations (Fig. 5C). The experimental values of the nematic order parameter were low at dextran sulfate concentrations below 0.06%, and increased in a concentration-dependent manner at higher dextran sulfate concentrations. On the other hand, the excentricity displayed a non-monotonous behavior, as it decreased with increasing dextran sulfate concentrations to reach a minimum al 0.06% dextran sulfate, and decreased in a concentration-dependent fashion at dextran sulfate concentrations above 0.06%. Notwithstanding the variability between measurements due to somewhat different numbers of cells in the microcolonies analyzed, the behavior of both parameters was highly reminiscent to that observed with the simulations at L*_0_ = 2.6 (Fig. 4), indicating that our mathematical model is at least a good qualitative predictor of microcolony shape and internal organization.

### Final remarks and wrap-up

In conclusion, we have developed a mathematical model to provide a description of the progress of a bacterial biofilm as it relates to the aspect ratio of the bacterial cells and the competition between bacterial growth and diffusion. The outcome of this competition is the emergence of two different growth regimes. Closed growth, characterized by small, compact, highly organized, and highly eccentric colonies, occurs preferentially when growth prevails over diffusion (i.e., at high Г values). In contrast, open growth, characterized by large, loose, poorly organized, nearly circular colonies, occurs preferentially in the opposite condition. Although we have purposefully restricted our model to two-dimensional growth on a flat surface, our current work is focused in extending this model to its application to three-dimensional biofilms.

### Computing methods

To model the kinetics of biofilm growth an individual particle with an initial aspect ratio L^*^_0_ (therefore representing a newborn cell) was seated in the center of simulation box at time t* = t/τ = 0, being t = σ^2^/D_0_ the time unit. The motion of the particles along the time has been modeled by means of Brownian Dynamics^24^ where, due to the lack of active motility of the particles, hydrodynamics interactions have not been considered. In this context, the equations of motion of the position and orientation of a particle *i* (defined by vector **r**_i_(t) and an unitary vector **ê**_**i**_(t)) are

1 $$\begin{array}{c}{{{\bf{r}}}_{{\rm{i}}}}^{||}({\rm{t}}+{\rm{\Delta }}{\rm{t}})={{{\bf{r}}}_{{\rm{i}}}}^{||}({\rm{t}})+{{\rm{D}}}^{||}{({{\rm{k}}}_{{\rm{B}}}{\rm{T}})}^{-1}{{{\bf{F}}}_{{\rm{i}}}}^{||}({\rm{t}}){\rm{\Delta }}{\rm{t}}+{({{\rm{2D}}}^{||}{\rm{\Delta }}{\rm{t}})}^{1/2}{{\rm{R}}}^{||}{\hat{\boldsymbol{e}}}_{{\rm{i}}}({\rm{t}})\\ {{{\bf{r}}}_{{\rm{i}}}}^{\perp }({\rm{t}}+{\rm{\Delta }}{\rm{t}})={{{\bf{r}}}_{{\rm{i}}}}^{\perp }({\rm{t}})+{{\rm{D}}}^{\perp }{({{\rm{k}}}_{{\rm{B}}}{\rm{T}})}^{-1}{{{\bf{F}}}_{{\rm{i}}}}^{\perp }({\rm{t}}){\rm{\Delta }}{\rm{t}}+{({{\rm{2D}}}^{\perp }{\rm{\Delta }}{\rm{t}})}^{1/2}{{\rm{R}}}^{\perp }{\rm{\Delta }}{\hat{\boldsymbol{u}}}_{{\rm{i}}}({\rm{t}})\\ {\hat{\boldsymbol{e}}}_{{\rm{i}}}({\rm{t}}+{\rm{\Delta }}{\rm{t}})={\hat{\boldsymbol{e}}}_{{\rm{i}}}({\rm{t}})+{{\rm{D}}}^{{\rm{\theta }}}{({{\rm{k}}}_{{\rm{B}}}{\rm{T}})}^{-1}{{\bf{T}}}_{{\rm{i}}}({\rm{t}})^{\prime} {\hat{\boldsymbol{e}}}_{{\rm{i}}}({\rm{t}}){\rm{\Delta }}{\rm{t}}+{({{\rm{2D}}}^{{\rm{\theta }}}{\rm{\Delta }}{\rm{t}})}^{1/2}{{\rm{R}}}^{{\rm{\theta }}}{\hat{\boldsymbol{u}}}_{{\rm{i}}}({\rm{t}})\end{array}$$

D^||^, D^⊥^ and D^θ^ are the parallel, perpendicular and rotational diffusion coefficients. These coefficients, that are calculated analytically^28^, depend on the aspect ratio of the particles, which is recalculated at each time step, and the diffusional coefficient D_0_. R^||^, R^⊥^ and R^θ^ are gaussian random numbers with null mean value and variance one. **û** _i_ is a unitary vector perpendicular to **ê**_i_. **F**_**i**_^||^, **F**_i_^⊥^ and are the parallel and perpendicular component of the total force over the particle i. **T**_i_ is the total torque acting over particle i. In our model these forces and torques are the result of the interaction with the other particles trough a Soft Repulsive Spherocylindrical potential^21^. By considering this short range interaction potential we assume that the only interaction between particles is due to exclude volume effects. This model is qualitatively similar to the Herztian frictional force used in other recent studies^15–20^. We have opted for the Soft Repulsive Potential due to its soft repulsion at small overlaps, and very strong repulsion at intermediate overlap, that helps prevent unrealistic particle overlaps.

Δt is the time-step in the simulation, that has been fixed as Δt = 10^−4^ t. In each time step the length of each particle grows as L(t + Δt) = L(t) + v_gr_ ·Δt, with v_gr_ the lengthening velocity. When the particle reaches its maximum aspect ratio L^*^_m_ = 2 L^*^_0_ it is split transversally into two identical particles with the initial aspect ratio L^*^_0_, the same orientation as the parent particle (**ê**_i_), and their centers located at **r**_1,2_ = r_0_ ± 0.5 (L_0_ + s)**ê**_i_, with **r**_0_ being the central position of the parent particle (Fig. 1A)

To systematize the results a relation between growth and diffusion has been defined as

2 $${\rm{\Gamma }}={{\rm{t}}}_{{\rm{dif}}}/{{\rm{t}}}_{{\rm{gr}}}$$

where ${{\rm{t}}}_{{\rm{dif}}}=0.25{{\rm{\sigma }}}^{2}/({{\rm{D}}}^{||}+{{\rm{D}}}^{\perp })$ is the time that an isolated particle of constant aspect ratio L^*^_0_ needs, in average, to be displaced a distance σ by brownian diffusion. ${{\rm{t}}}_{{\rm{gr}}}=({{\rm{L}}}_{{\rm{0}}}+{\rm{\sigma }})/{{\rm{v}}}_{{\rm{gr}}}$ is the time that a bacteria needs to reach the aspect ratio L^*^_m_ from the aspect ratio of a newborn bacterium L^*^_0_. In our simulations, we have fixed the value of D_0_ = 0. 1 and adjust the value of v_gr_ to obtain the desired value of Γ. Preliminary trials show that for a given value of Г the results obtained are independent of the values of v_gr_ and D_0_.

To evaluate the shape and internal structures of the particle clusters, the eccentricity Ф and nematic order parameter S_2_ were calculated. For a colony with N particles, Ф is calculated by diagonalization of the inertia tensor

3 $${{\rm{I}}}_{{\rm{i}},{\rm{j}}}=1/{\rm{N}}({{\rm{\Sigma }}}_{{\rm{k}}={\rm{1}},{\rm{N}}}{{\rm{\delta }}}_{{\rm{ij}}}({{\rm{\Sigma }}}_{{\rm{m}}={\rm{i}},{\rm{j}}}{{{\rm{r}}}^{2}}_{{\rm{k}}}({\rm{m}}))-{{\rm{r}}}_{{\rm{k}}}({\rm{i}}){{\rm{r}}}_{{\rm{k}}}({\rm{j}}))$$

with N the number of particles and i and j represent the coordinates x and y. Then, Ф = (1 − I’_x_/I’_y_)^1/2^, with I’_x_ and the lower and I’_y_ higher eigenvalues of **I**. This magnitude provides information about the shape of the particle clusters, with values close to zero for circular clusters, and values between zero and one for elliptic clusters. The nematic order parameter S_2_ is calculated with the standard procedure of diagonalize a symmetric tensor traceless build with the orientation vectors of all the particles^24,25^.

The coverage profile is defined as g(r) = A_o_(r)/A (r), where A(r) is the area of a annulus centered in the center of mass of the cluster, with internal and external radius r and r + dr. A_o_(r) is the area of this annulus covered by particles. We have calculated this function generating a high number of random points in the annulus considered, evaluating g(r) as the fraction of these points that are inside a particle divided by the total number of points in the annulus.

In general, the numerical results from computer simulations presented in this article have been obtained averaging over ten realizations of the full biofilms, starting in all the cases with a different seed for the random number generator.

### Microbiological methods

For image analysis of biofilm microcolonies, cells of *P*. *putida* KT2442^29^ and its ∆*fleQ mutant* derivative MRB52 (Lorena Jiménez-Fernández and Fernando Govantes, unpublished) were grown aerobically in LB medium overnight at 30 °C, diluted 100-fold and re-grown in the same conditions to an OD_600_ of ~0.25. Dilutions of this culture (100 µl) were transferred to flat bottom polystyrene microtiter plate wells (Corning) and incubated for 5 to 30 minutes at room temperature to allow attachment. The wells were subsequently washed three times with LB and overlayed with 100 µL LB. When required, dextran sulfate (Mw >500,000, Sigma-Aldrich) was added to LB at this step. Surface-associated cells were further incubated at room temperature without shaking for different time periods prior to microscope observation. Microscope images and time lapse movies of *P*. *putida* microcolonies were captured using an inverted Leica DMI4000B microscope coupled to a digital camera using the 40X objective in the phase contrast mode and the manufacturer’s software.

Microscopy images were analyzed automatically using ImageJ v1.49 m to determine the position, orientation and shape of the bacteria of the colonies studied. After background subtraction, a brightness threshold was empirically determined to differentiate individual cells. Particles smaller than bacterial cells identified by this method were automatically discarded by filtering out objects below an area of 0.002 arbitrary units. Parameters for characterization of the individual cells or the microcolonies were calculated from the images as described above for the simulations.

## Electronic supplementary material

Supplementary Information

Supplementary movie S1. Time-lapse movie of early microcolony development in KT2442.

Supplementary movie S2. Time-lapse movie of early microcolony development in MRB52.

Supplementary movie S3. Time-lapse movie of surface motility in individual KT2442 cells in the absence of dextran sulfate.

Supplementary movie S4. Time-lapse movie of surface motility in individual KT2442 cells in the presence of dextran sulfate.

Supplementary movie S5. Time-lapse movie of surface motility in individual MRB52 cells in the absence of dextran sulfate.

Supplementary movie S6. Time-lapse movie of surface motility in individual MRB52 cells in the presence of dextran sulfate.

## References

[1] Costerton JW, Lewandowski Z, Caldwell DE, Korber DR, Lappin-Scott HM (1995). Microbial biofilms. Annu. Rev. Microbiol..

[2] Costerton JW, Stewart PS, Greenberg EP (1999). Bacterial biofilms: a common cause of persistent infections. Science.

[3] O’Toole GA, Kaplan HB, Kolter R (2000). Biofilm formation as microbial development. Annu. Rev. Microbiol..

[4] Monds RD, O’Toole GA (2009). The developmental model of microbial biofilms: ten years of a paradigm up for review. Trends Microbiol..

[5] Peyton BM, Characklis WG (1995). Microbial biofilms and biofilm reactors. Bioprocess Technol..

[6] Davey ME, O’Toole GA (2000). Microbial biofilms: from Ecology to Molecular Genetics. Microbiol. Mol. Biol. Rev..

[7] Shehata TE, Marr AG (1971). Effect of Nutrient Concentration on the Growth of Escherichia coli. J. Bacteriol.

[8] Richardson IG (1999). The nature of C-S-H in hardened cements. Cem. Con. Res..

[9] Pellenq RJ-M (2009). A realistic molecular model of cement hydrates. Proc Natl Acad Sci USA.

[10] Berger T, Anta JA, Morales-Flórez V (2013). Surface Properties of Anatase TiO_2_ Nanowire Films Grown from a Fluoride-Containing Solution. Chem PhysChem.

[11] Wang Q, Zhang T (2010). Review of mathematical models for biofilms. Solid State Comm..

[12] Horn H, Lackner S (2014). Modeling of Biofilm Systems: A Review. Adv Biochem Eng Biotechnol.

[13] Kreft J-U, Wimpenny JWT (2001). Effect of EPS on biofilm structure and function as revealed by an individual-based model of biofilm growth. Water Sci Technol.

[14] Picioreanu C, Kreft JU, van Loosdrecht MCM (2004). Particle-Based multidimensional multispecies biofilm model. Appl Environ Microbiol.

[15] Volfson D, Cookson S, Hasty J, Tsimring LS (2008). Biomechanical ordering of dense cell populations. Proc Natl Acad Sci USA.

[16] Grant MAA, Waclaw B, Allen RJ, Cicuta P (2017). The role of mechanical forces in the planar-to-bulk transition in growing *Escherichia coli* microcolonies. J. R. Soc. Interface.

[17] Ghosh P, Mondal J, Ben-Jacob E, Levine H (2015). Mechanically-driven phase separation in a growing bacterial colony. Proc Natl Acad Sci USA.

[18] Winkle JJ, Igoshin OA, Bennet MR, Josić K, Ott W (2017). Modelling mechanical interactions in growing populations of rod-shaped bacteria. Phys Biol.

[19] Farrell FD, Gralka M, Hallatschek O, Waclaw B (2017). Mechanical interactions in bacterial colonies and the surfing probability of beneficial mutations. J R So. Interface.

[20] You, Z., Pearce, D. J. G. Sengupta, A. & Giomi, L. Geometry and mechanics of micro-domains in growing bacterial colonies. *arXiv* **1703**, 04504 [cond-mat.soft] (2017).

[21] Cuetos A, Martínez-Haya B (2015). Liquid crystal phase diagram of soft repulsive rods and its mapping on the hard repulsive reference fluid. Mol Phy..

[22] Verstraeten N (2008). Living on a surface: swarming and biofilm formation. Trends Microbiol..

[23] Löwen H (1994). Brownian dynamics of hard spherocylinders. Phys Rev E.

[24] Allen MP, Evans GT, Frenkel D, Mulder BM (1993). Hard convex body fluids. Adv Chem Phys.

[25] Eppenga R, Frenkel D (1984). Monte Carlo study of the isotropic and nematic phases of infinitely thin hard platelets. Mol Phys.

[26] Jiménez-Fernández A (2016). Complex Interplay between FleQ, cyclic diguanylate and multiple σ factors coordinately regulates flagellar motility and biofilm development in *Pseudomonas putida*. PLoS One.

[27] Antoniou E, Tsianou M (2012). Solution properties of dextran in water and in formamide. J Appl Polym Sc..

[28] Shimizu H (1962). Effect of Molecular Shape on Nuclear Magnetic Relaxation. J Chem Phys.

[29] Franklin FC, Bagdasarian M, Bagdasarian MM, Timmis KN (1981). Molecular and functional analysis of the TOL plasmid pWWO from *Pseudomonas putida* and cloning of genes for the entire regulated aromatic ring meta cleavage pathway. Proc Natl Acad Sci USA.

