# Supplementary material for: Computer simulation study of early bacterial biofilm development
Source: Sci Rep. 2018 Mar 28;8:5340. doi: 10.1038/s41598-018-23524-x (PMC5871757; doi:10.1038/s41598-018-23524-x)
Supplement: Supplementary file 1 — Supplementary Information [file 41598_2018_23524_MOESM1_ESM.pdf]

SUPPLEMENTARY INFORMATION

Computer simulation study of early bacterial biofilm development.

Rafael D. Acemel, Fernando Govantes and Alejandro Cuetos*

***Correspondence to:** acuemen@upo.es

This document include:

Fig. S1

Legends to supplementary movies

SUPPLEMENTARY FIGURES

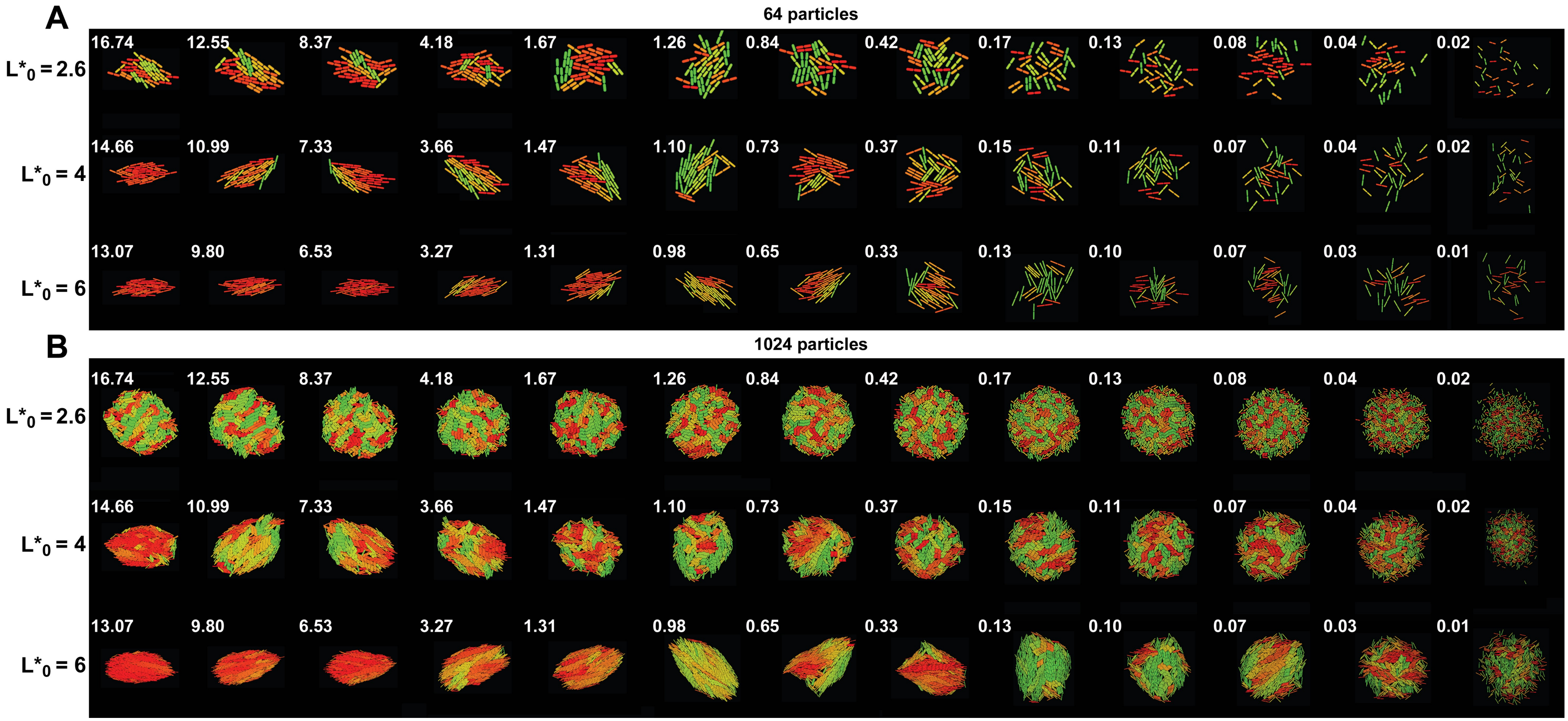

Supplementary Figure S1. Typical results from computer simulations. Snapshots showing the shape and internal structure of clusters containing 64 (A) or 1024 (B) particles with aspect ratios L^*_0 = 2.6, 4 or 6 at all Γ values used in the simulations. Particle color indicates orientation in a scale ranging from green (vertical) to red (horizontal).

LEGENDS TO SUPPLEMENTARY MOVIES

Supplementary movie S1. Time-lapse movie of early microcolony development in KT2442. Surface growth of a single KT2442 cell associated to a polystyrene microtiter well surface and overlayed with LB was monitored at 2-minute intervals for 270 minutes.

Supplementary movie S2. Time-lapse movie of early microcolony development in MRB52. Surface growth of a single MRB52 cell associated to a polystyrene microtiter well surface and overlayed with LB was monitored at 2-minute intervals for 270 minutes.

Supplementary movie S3. Time-lapse movie of surface motility in individual KT2442 cells in the absence of dextran sulfate. Surface motility of a individual KT2442 cells associated to a polystyrene microtiter well surface and overlayed with LB was monitored at 10-second intervals for 10 minutes.

Supplementary movie S4. Time-lapse movie of surface motility in individual KT2442 cells in the presence of dextran sulfate. Surface motility of individual KT2442 cells associated to a polystyrene microtiter well surface and overlayed with LB supplemented with 0.25% w/v dextran sulfatewas monitored at 10-second intervals for 10 minutes.

Supplementary movie S5. Time-lapse movie of surface motility in individual MRB52 cells in the absence of dextran sulfate. Surface motility of individual MRB52 cells associated to a polystyrene microtiter well surface and overlayed with LB was monitored at 10-second intervals for 10 minutes.

Supplementary movie S6. Time-lapse movie of surface motility in individual MRB52 cells in the presence of dextran sulfate. Surface motility of individual MRB52 cells

associated to a polystyrene microtiter well surface and overlayed with LB supplemented with 0.25% w/v dextran sulfate was monitored at 10-second intervals for 10 minutes.
